# The Value of Cerebral CT Angiography with Low Tube Voltage in Detection of Intracranial Aneurysms

**DOI:** 10.1155/2015/876796

**Published:** 2015-02-01

**Authors:** Kun Tang, Rui Li, Jie Lin, Xiangwu Zheng, Ling Wang, Weiwei Yin

**Affiliations:** ^1^Department of Radiology, The First Affiliated Hospital of Wenzhou Medical University, Wenzhou, Zhejiang 325000, China; ^2^Department of Nuclear Medicine, Wenzhou Medical University, Wenzhou, Zhejiang 325000, China

## Abstract

*Objective*. The aim of this study is to investigate the value of cerebral CT angiography (CTA) with low tube voltage in detection of intracranial aneurysms. * Materials and Methods*. A total of 294 consecutive patients with spontaneous subarachnoid hemorrhage (SAH) were enrolled in this study and randomly assigned into conventional voltage CTA (C-CTA) group and low voltage CTA (L-CTA) group. The objective and subjective image qualities were analyzed and compared between C-CTA and L-CTA groups. With the results of 3D-DSA as “gold standard,” the sensitivity, specificity, and accuracy of C-CTA and L-CTA in diagnosis of aneurysms were calculated and compared with each other. * Results*. Compared with group C-CTA, the CT dose index volume (CTDIvol) of group L-CTA reduced by 35.65%. There were no significant differences between C-CTA and L-CTA groups regarding objective and subjective image qualities. The sensitivity, specificity, and accuracy of L-CTA in diagnosis of aneurysms were 95.16%, 99.72%, and 99.42%, respectively. There were no significant differences in sensitivity, specificity, and accuracy between the C-CTA and L-CTA groups. * Conclusion*. The value of cerebral CTA with 100 kV low tube voltage in detection of intracranial aneurysms is significant, and it should be recommended as a routine scan method.

## 1. Introduction

Spontaneous subarachnoid hemorrhage (SAH) is caused by rupture of an intracranial aneurysm in 80–90% [1, 2] of cases. The mortality for untreated aneurismal SAH is up to 43–67% [3–5] in the first month. Therefore, early diagnosis and early treatment are the most effective way to prevent the death of patients. Recently, computed tomography angiography (CTA) technology is playing an increasing role in the screening of patients suspected of having intracranial aneurysms, but it also has the potential to lead to an increase in radiation dose owing to the routine use of thinner sections and the extended volume of acquisition. Although magnetic resonance angiography (MRA) is an alternative nonradiation technique in diagnosis of intracranial aneurysms, the main disadvantages of this technique are the prolonged acquisition time and the artifacts due to flow phenomena and patient motion. A recent meta-analysis indicates that sensitivity of MRA has become comparable with that of CTA in the diagnosis of intracranial aneurysms, but specificity of MRA seems inferior compared with CTA [6]. Therefore, CTA has widely been used as a routine primary tool in diagnosis of intracranial aneurysms, and the concern regarding radiation dose in CT scan has been increasing, especially the potential harm to the brain [7].

However, there are few reports regarding low radiation dose during neuroimaging, especially the CTA with low tube voltage in detection of intracranial aneurysms. Most of the available studies using low dose CTA focus on great vessels, such as carotid artery [8] and pulmonary artery [9]. Even in those articles referring to neurovascular imaging with low tube voltage, the results were inconsistent and the cases involved in those studies were limited [10–13]. In this study, a large sample was enrolled and assigned into conventional voltage CTA (C-CTA) group and low voltage CTA (L-CTA) group. The aim of this study is to investigate the value of CTA with low tube voltage in detection of intracranial aneurysms by comparing image qualities, radiation dose, and detection accuracy of aneurysms between C-CTA group and L-CTA group, with three-dimensional digital subtraction angiography (3D-DSA) as the reference standard.

## 2. Materials and Methods

The institutional review board approved this study, and informed consent was obtained from all participating patients.

### 2.1. Patients

Between October 2010 and May 2012, a total of 294 consecutive patients (130 males, 164 females; mean age 56.89 ± 13.30 years) with SAH were enrolled in this prospective study. The inclusion criteria were that patients have clinical evidence of intracranial aneurysms and are able to undergo both CTA and DSA. The exclusion criteria were history of allergy to iodine-containing contrast medium, serious cardiac renal insufficiency, and pregnancy.

The patients who were enrolled in this study were randomly assigned into C-CTA group and L-CTA group. The C-CTA group included 148 patients (64 males, 84 females; mean age 56.66 ± 13.02 years) and the L-CTA group included 146 patients (66 males, 80 females; mean age 57.13 ± 13.62 years). All of the patients underwent both 64-slice CTA and 3D-DSA within 3 days successively.

### 2.2. Study Protocols

#### 2.2.1. CTA Protocol

All imaging procedures were performed using a 64-row multislice CT system (GE LightSpeed, 64-row multislice CT). The CTA was initiated 18–20 seconds after the start of infusion of nonionic iodinated contrast material (iohexol 350 mgI/mL) from dorsal vein or antecubital vein. Nonionic iodinated contrast material was injected with a powered injector at a rate of 4 mL/s. The volume of nonionic iodinated contrast material in each study was generally 60–80 mL. Scanning range is from basis cranii to calvaria.

The CTA data acquisition was performed according to the following protocol: tube voltage of 120 kV (C-CTA) or 100 kV (L-CTA); tube current of 380 mA; slice thickness of 5.0 mm; section interval of 5.0 mm; collimation of 64 × 1.25 mm; rotation time of 0.6 s; pitch of 0.562; FOV of 25 cm; matrix of 512 × 512; reconstruction thickness of 1.25 mm; and reconstruction interval of 0.6 mm.

The review of the CT images was processed by a workstation (ADW Workstation 4.3) to acquire MRP, MIP, and VR algorithms in each case.

#### 2.2.2. DSA Protocol

All DSA examinations were performed with femoral catheterization by Seldinger technique with a DSA system (Philips Allura Xper FD20, Netherlands). Typically, nonionic iodinated contrast material (iohexol 350 mgI/mL) was used in all cases. Standard injection rates and volumes were as follows: 5 mL/s for 8 mL for common carotid artery and 4 mL/s for 7 mL for vertebral artery. The parameters were as follows: rotation angle: 0–2400; rotational speed: 550/s; picking rate: 30 frames/s; image matrix: 1024 × 1024; image reconstruction matrix: 256 × 256; and exposure lag: 2 s. The 3D-DSA injection rates and volumes were as follows: 5 mL/s for 25 mL for common carotid artery and 3 mL/s for 12 mL for vertebral artery. The review of the DSA image data was processed by a workstation (Philips Integris 3D RA Release 4.3) to acquire 3D-DSA reconstructed images, such as VRD and SSD algorithms.

### 2.3. Image Analysis

#### 2.3.1. Objective Image Quality of CTA

The CT attenuation values were measured on axial images using a manually defined circular region of interest (ROI) with diameter of 2–10 mm. The measurement locations were as follows: basal artery (BA), left and right side of the internal carotid artery (ICA) of intracranial segment, the anterior cerebral artery (ACA), middle cerebral artery (MCA), and posterior cerebral artery (PCA). The CT values were measured three times at different segment of each vessel, using the average for the vascular CT value. Taking occipital lobe parenchyma (OLP) as vascular background, image noise is the standard deviation (SD) of the attenuation values of the background. All CTA images measurement was independently performed by an experienced radiologist.

Contrast-to-noise ratio (CNR) and signal-to-noise ratio (SNR) are calculated as follows: CNR = (ROIa − ROIb)/SD; SNR = ROIa/SD, where ROIa and ROIb are the CT numbers of the vessel region of interest and of the background region of interest, respectively.

#### 2.3.2. Subjective Image Quality of CTA

For the subjective assessment of CTA image quality, two experienced observers who were blind to each set of scanning parameters were asked to evaluate all CTA images independently. The arterial margin, depiction of small arterial details, venous contamination, side-branches, adjacent perforators, the whole image quality, and the diagnostic confidence were assessed, respectively, by the use of a 5-point score system. The scoring criteria details are shown in Table 1.

### 2.4. Aneurysm Analysis

All DSA and CTA images were evaluated by consensus of two experienced neuroradiologists. The observation and analysis of the aneurysm on CTA and 3D-DSA were evaluated at 13 standard vessels [14, 15]: (1) the left and right vertebral artery of intracranial segment, (2) basilar artery, (3) posterior communicating artery, (4) left and right posterior cerebral artery, (5) left and right internal carotid artery of intracranial segment, (6) the left and right middle cerebral artery, (7) left and right anterior cerebral artery, and (8) anterior communicating artery.

If an aneurysm was considered present, the largest diameter and the size of the aneurysm neck of each aneurysm on CTA and 3D-DSA images were measured by one experienced neuroradiologist. The largest diameter of each aneurysm was measured and graded as ≥5 mm, 3–5 mm, or ≤3 mm. In addition, the anatomical relationship between aneurysm and adjacent structures and the feeding artery of the aneurysm was analyzed.

### 2.5. Radiation Dose

We used the CT dose index volume (CTDIvol, mGy) provided by the manufacturer for estimation of radiation dose. Effective dose (ED, mSv) was calculated according to dose-length product (DLP, mGy·cm) and the tissue weighting factor *k* (mSv × mGy^−1^ × cm^−1^) commended by the European Commission (0.0021) [16]. The calculated formula is ED = *k* × DLP.

### 2.6. Statistical Analysis

We used two-tailed Student's* t*-test to evaluate differences in scan length, image objective quality, and radiation dose between group C-CTA and group L-CTA. For subjective assessment, Wilcoxon's signed-rank test was used to analyze the differences between the two CTA groups. The sensitivity, specificity, and accuracy of diagnosis were calculated by using chi-square test. The largest diameter of aneurysm and the size of aneurysm neck in both CTA groups were compared with DSA using correlation analysis. Interobserver variation was assessed using Cohen kappa statistics. Kappa values less than 0.20 indicated poor agreement; 0.21–0.40, fair agreement; 0.41–0.60, moderate agreement; 0.61–0.80, good agreement; and 0.81–1.00, excellent agreement. All statistical analyses were performed with a commercially available software package (SPSS, version 17.0) and a* P* value of less than 0.05 was considered to be statistically significant.

## 3. Results

A total of 294 patients (130 males, 164 females) were enrolled in this study and underwent CTA and 3D-DSA within 3 days successfully. The average scan length of C-CTA and L-CTA was 16.73 ± 1.35 cm and 16.59 ± 1.03 cm, respectively. The difference of scan length between the two groups was not statistically significant (*P* = 0.720).

### 3.1. Radiation Dose

The results of radiation dose including CTDIvol, DLP, and ED of group C-CTA and group L-CTA are shown in Table 2. Compared with the group C-CTA, the CTDIvol, DLP, and ED of group L-CTA were reduced by 35.65%, 35.27%, and 35.35%, respectively. The differences of average DLP and ED between the two groups were statistically significant (*P* = 0.000).

### 3.2. Image Quality Results

The mean vessel attenuation of group L-CTA was increased by approximately 19%–22% compared with the group C-CTA. The difference between the two groups was statistically significant (*P* = 0.000). At the same time, the contrast value between vessels and cerebral parenchyma at low voltage group was also substantially higher than that of standard voltage group (*P* = 0.000). Although the noise level was markedly higher at group L-CTA, the calculated CNRs and SNRs showed no differences between the two groups (CNRs: *P* = 0.509, 0.596, 0.833, 0.933, and 0.868; SNRs: *P* = 0.285, 0.367, 0.744, 0.590, and 0.737).

There were no significant differences between C-CTA and L-CTA groups regarding scores for arterial margin, depiction of small arterial details, venous contamination, side-branches, adjacent perforators, the whole image quality, and the diagnostic confidence (*P* = 0.121, 0.325, 0.233, 0.112, 0.233, 0.413, and 0.883). Using Cohen kappa statistics, the interobserver agreement in regard to subjective assessment of image quality was 0.77, 0.73 (kappa > 0.60), which meant that the observation had good consistency and high reliability.

### 3.3. Aneurysm Diagnosis Results

In all 294 cases, a total of 273 aneurysms were identified by 3D-DSA in 243 patients. Solitary aneurysm was found in 215 cases, while multiple aneurysms were found in 28 cases, including 26 patients with two aneurysms and 2 patients with 3 aneurysms. The mean diameter of the aneurysms was 5.26 ± 2.64 mm (range 1.5 to 18.3 mm). The distribution of aneurysms according to the size was 123 of ≥5 mm; 114 of 3 to 5 mm; and 36 of <3 mm. The mean size of aneurysms neck was 2.99 ± 1.14 mm (range 0.7 to 9.4 mm).

**Figure 1:**
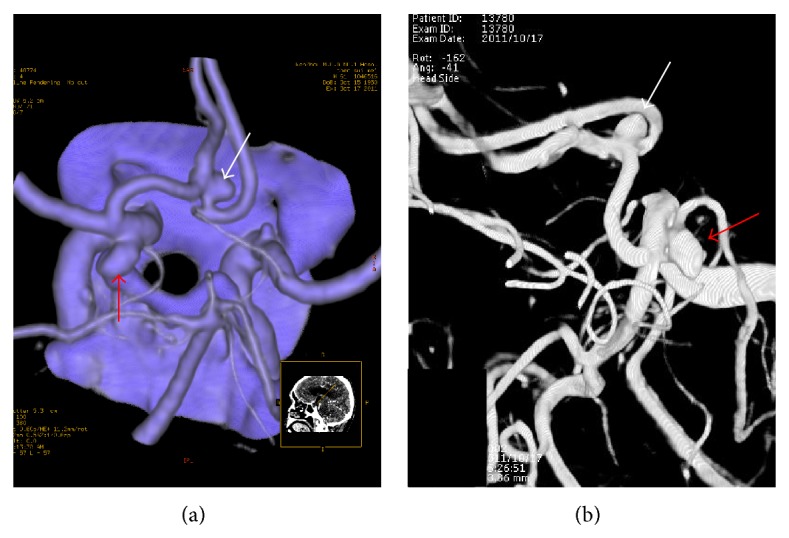
Images from a 61-year-old woman with SAH. L-CTA scanning shows an aneurysm ((a), white arrow) at anterior communicating artery and an aneurysm ((a), red arrow) at posterior communicating artery. 3D-DSA depicts the aneurysms at the same locations ((b), white and red arrows). The maximum diameter and neck sizes of anterior communicating artery aneurysm were 7.32 mm and 3.40 mm ((b), white arrow); and those of posterior communicating artery aneurysm were 5.44 mm and 3.86 mm ((b), red arrow).

**Figure 2:**
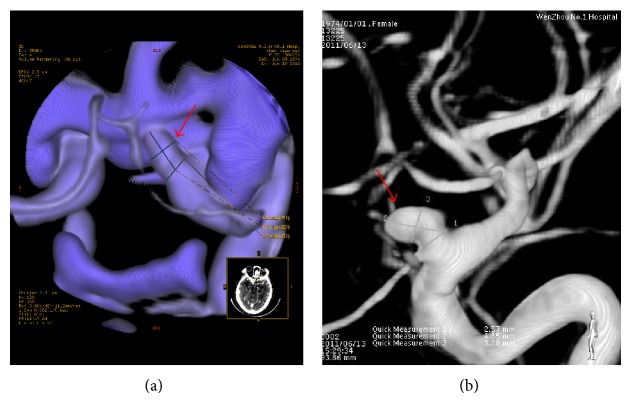
Images from a 37-year-old woman with SAH. The L-CTA (a) and 3D-DSA (b) showed the same aneurysm at the same location. The maximum diameter of aneurysm and neck sizes in C-CTA (a) and 3D-DSA (b) were 4.5, 2.7 mm and 4.85, 2.57 mm, respectively (red arrow).

Compared with the 3D-DSA reference standard, the accuracy of C-CTA and L-CTA in detection of intracranial aneurysms is shown in Table 3 and Figures 1 and 2. Three aneurysms were misdiagnosed and nine aneurysms were missed with C-CTA, while five aneurysms were misdiagnosed and six aneurysms were missed with L-CTA. Compared with 3D-DSA, the accuracy of C-CTA and L-CTA in diagnosis of aneurysms was not statistically different. Thesensitivity, specificity, and accuracy in the C-CTA and L-CTA were 93.96%, 99.83%, and 99.38% and 95.16%, 99.72%, and 99.42%, respectively. There were no statistically significant differences between the two groups. For aneurysms of ≥5 mm, 3 to 5 mm, and <3 mm, the diagnostic results of C-CTA and L-CTA were shown in Table 4. The differences of the two groups in detection of different size of aneurysms were not statistically significant (*P* = 1.0).

The mean maximum diameter and mean neck size of aneurysms in group C-CTA and group L-CTA were 5.53 ± 2.55 mm (95% CI: 5.11–5.95), 2.97 ± 1.24 mm (95% CI: 2.77–3.17) and 5.70 ± 2.60 mm (95% CI: 4.76–6.64), 3.03 ± 1.04 mm (95% CI: 2.53–3.53), respectively. The differences of the mean maximum diameter and mean neck size between CTA and 3D-DSA were not statistically significant (C-CTA/3D-DSA: *P* = 0.846, 0.745; L-CTA/3D-DSA: *P* = 0.642, 0.708). In both groups, there were significant correlations for maximum aneurysm diameter and aneurysm neck diameter measurement between CTA and 3D-DSA (*r* = 0.975, *r* = 0.954; *r* = 0.940, *r* = 0.931).

## 4. Discussion

At present, traditional DSA examination still remains the gold standard for detection of aneurysm, but it has several limits: it is invasive and time-consuming, the operating technology is complicated, and it has about 1–2.3% of surgical complications [17]. As a noninvasive examination, the value of cerebral CTA in diagnosis of intracranial aneurysm is widely accepted by clinic, owing to its features of being noninvasive, simple, rapid, and accurate. But it also has the potential to lead to an increase in radiation dose, owing to the routine use of thinner sections and the extended volume of acquisition. Previous literature reported that the effective dose produced by one routine brain CT scan is 0.9–4.0 mSv, while the radiation dose which resulted from cerebral CTA is much greater. If a dual-phase or a subtraction angiography scan is performed, the radiation dose is 2 to 3 times more than that of single phase cerebral CTA scan [18]. Therefore, concerns regarding a reduction in radiation dose have been recently raised during cerebral CTA acquisitions [19].

Cerebral CTA scan with lower tube voltage can greatly reduce the radiation dose. In our study, reduction of tube voltage from 120 KV to 100 KV while keeping other parameters the same led to an approximate 35% decrease in radiation dose. Sun et al. [12] suggested that a tube voltage of 80 KV resulted in an approximate 69.73% reduction in radiation dose compared with radiation exposure at 120 KV. However, the main drawback of the low tube voltage technique is the increase of image noise caused by the reduced photon flux. Some studies [11] suggested that increase of noise will affect the appearance of small diameter arteries, especially posterior circulation arterial aneurysms. In our study, there were no significant differences in objective and subjective image qualities between two groups. Furthermore, the aneurysm detectable rate of L-CTA group (41/48) is higher than that of C-CTA group (38/49). The analysed reasons are as follows: (1) the cerebral CTA scan with low tube voltage can significantly increase the vascular enhancement value, so it can improve the appearance of aneurysm itself; (2) the contrast between aneurysm and the surrounding parenchyma can be also increased, which will make up for the noise increase to a certain extent.

Without degradation of image quality, the value of low tube voltage CTA scan in detection of intracranial aneurysms was high. With the results of 3D-DSA as “gold standard,” the sensitivity, specificity, and accuracy of C-CTA and L-CTA in diagnosis of aneurysms were 93.96%, 99.83%, and 99.38% and 95.16%, 99.72%, and 99.42%, respectively. There were no significant differences in sensitivity, specificity, and accuracy between the C-CTA and L-CTA groups, which were consistent with previous results [12].

Compared with the conventional DSA, CT image has relatively low spatial resolution. Therefore, the diagnostic accuracy of microaneurysms is much lower. Previous literatures [20, 21] reported that the CTA diagnostic accuracy of aneurysm with diameter <3 mm was 74.1% to 84.0%. In our study, the CTA diagnostic accuracy of aneurysms with diameter ≥3 mm achieved above 95%, while the <3 mm aneurysms' detection rate was low (C-CTA: 78.26%; L-CTA: 76.92%). However, there were no statistically significant differences in diagnostic accuracy between the two groups. This indicates that the ability of detection of microaneurysm will not be affected by lower tube voltage CTA scan. The reasons of false-positive and false-negative results were based on diagnostic experience and improper operation or were due to aneurysm's secluded location or were affected by vascular malformation. The missed aneurysms in both C-CTA and L-CTA groups can all be revealed on CTA images after retrospective reconstruction and analysis.

In conclusion, as an effective technique of reducing CT radiation dose, cerebral CTA with low tube voltage provides qualified image quality and high diagnostic value in detection of intracranial aneurysms. Therefore, it is a promising alternative for evaluating intracranial aneurysms and should be recommended as a routine scan method.

## Figures and Tables

**Table 1:** Subjective image quality assessment.

Evaluation items	5 points	4 points	3 points	2 points	1 point
Arterial margin	Smooth	Mild rough	Moderate Rough	Rough	Extensive Rough
Depiction of small arterial details	Best	Better	General	Bad	Worse
Venous contamination	Least	Less	General	Much	More
Side-branches	Clear	Moderate clear	Mild clear	Rough	Unclear
Perforators	Clear visibility	Moderate visibility	Mild visibility	Hardly visible	Invisible
The whole image quality	Best	Better	General	Bad	Worse
Diagnostic confidence	Sufficient	Less sufficient	General	Insufficient	Unable to diagnose

**Table 2:** The comparative results of radiation dose between C-CTA and L-CTA groups.

Radiation dose	Group C-CTA	Group L-CTA	*t*	*P*
CTDIvol (mGy)	55.73	35.86	⋯	⋯

Scan length (cm)	16.73 ± 1.35	16.59 ± 1.03	0.37	0.720
DLP (mGy·cm)	932.55 ± 112.35	594.76 ± 102.34	18.24	0.000
ED (mSv)	1.96 ± 0.45	1.25 ± 0.39	18.24	0.000

**Table 3:** Accuracy of C-CTA and L-CTA in the detection of intracranial aneurysms.

Group	True positive	False positive	False negative	True negative	Sensitivity	Specificity	Accuracy
C-CTA	140	3	9	1777	93.96%	99.83%	99.38%
L-CTA	118	5	6	1771	95.16%	99.72%	99.42%

**Table 4:** The results of C-CTA and L-CTA in detection of various sizes of aneurysms.

Size of aneurysms	C-CTA		L-CTA		Detection rate		Chi-square value	*P* value
	Detection number	Missing number	Detection number	Missing number	C-CTA	L-CTA		
≥5 mm	62	1	59	1	98.41%	98.33%	⋯	1.0
3~5 mm	60	3	49	2	95.24%	96.08%	0.000	1.0
<3 mm	18	5	10	3	78.26%	76.92%	0.000	1.0
